# A Novel Uterus‐Sparing Technique for Placenta Accreta Spectrum (PAS), at a Tertiary Center

**DOI:** 10.1155/ogi/1224673

**Published:** 2026-07-30

**Authors:** Samir Ghourab, Nada Alsahan, Osama Alomar, Reem Labani

**Affiliations:** ^1^ King Faisal Specialist Hospital and Research Centre, Riyadh, Saudi Arabia, kfshrc.edu.sa

## Abstract

**Objective:**

To present and evaluate the outcomes of a novel targeted devascularization technique for managing a placenta accreta spectrum (PAS).

**Design:**

Prospective observational study.

**Setting:**

The main tertiary care center in Saudi Arabia.

**Population:**

Sixty‐seven referred pregnant women with a previous cesarean section scar, placenta previa, and sonographic diagnosis of PAS.

**Methods:**

Patients with PAS underwent targeted devascularization of the lateral sides of the uterus between cervix, and round ligament insertion at the time of caesarean delivery. Primary outcomes included uterine preservation rate and maternal morbidity. Secondary outcomes were transfusion requirements, surgical time, maternal and neonatal outcomes, estimated blood loss, transfusion rate, and bladder injury.

**Results:**

Among 67 patients, conservative surgery was successful in 95.5%, with a mean blood loss of 1.57 ± 0.81 L and 82.1% requiring blood transfusion. Bladder injury occurred in 22.4% and was managed intraoperatively. Surgical performance improved over time with reduced transfusion requirements (*p* = 0.004) and shorter operative times (*p* = 0.002). No long‐term complications or a maternal death were recorded.

**Conclusions:**

Targeted devascularization of the lateral side of the uterus is a safe and effective uterus‐sparing approach for PAS, associated with low morbidity and improved operative efficiency over time.

## 1. Introduction

Placenta accreta spectrum (PAS) is a life‐threatening obstetric condition characterized by abnormal trophoblastic invasion into the myometrium and beyond, It is a spectrum disorder ranging from abnormal adherence to deep invasive placental tissues [1] incidence has increased globally in parallel with rising caesarean delivery rates, now representing a major cause of obstetric hemorrhage, maternal morbidity, and emergency hysterectomy [2].

Conventional management often involves caesarean hysterectomy, but various conservative approaches have been proposed to preserve the uterus, particularly in young women desiring future fertility, management strategies include focal myometrial resection and conservative management leaving the placenta in situ after delivery with or without adjunctive measure such as arterial embolization or planned delayed hysterectomy.

However, these are often associated with significant complications and variable success rates [3].

In response to these challenges, we developed a novel uterus‐sparing technique involving target devascularization of uterine part which is likely to cause massive hemorrhage during removal of placenta in patient with PAS, this technique have been adopted for all patient with PAS from 2016 until 2025 after being refined and modified during the previous 10 years prior to 2016 in order to reduce blood loss to minimal and reduce surgical morbidity. This study presents our experience using this technique in a prospective cohort of patients with PAS, aiming to evaluate its safety, efficacy, and clinical outcomes.

## 2. Methods

This prospective observational study was conducted at the main tertiary hospital in Saudi Arabia. Patients referred electronically with detailed medical reports and sonographic imaging of the placenta, patients who are accepted for conservative surgical management should have previous cesarean section, placenta previa, and sonographic evidence of PAS which include bridging vessels, myometrium less than 1 mm thickness, loss of retroplacental clear zone, and placental bulge with or without lacunae [4], Ethical approval for this study was obtained from the Institutional Review Board of King Faisal Specialist Hospital and Research Centre, Riyadh, Saudi Arabia (RAC number: 2131035). Informed consent was obtained from all participants prior to inclusion in the study. Written permission was also secured from the women whose placental images are included in this article.

All patient are scanned and managed by the same expert team in the management of PAS headed by the first author, admissions were arranged at 30 to 34 gestational age and surgery at 35–36 weeks gestation or earlier in case of documented uterine contraction, vaginal bleeding, or for fetal indication.

### 2.1. Surgical Procedure

**FIGURE 1:**
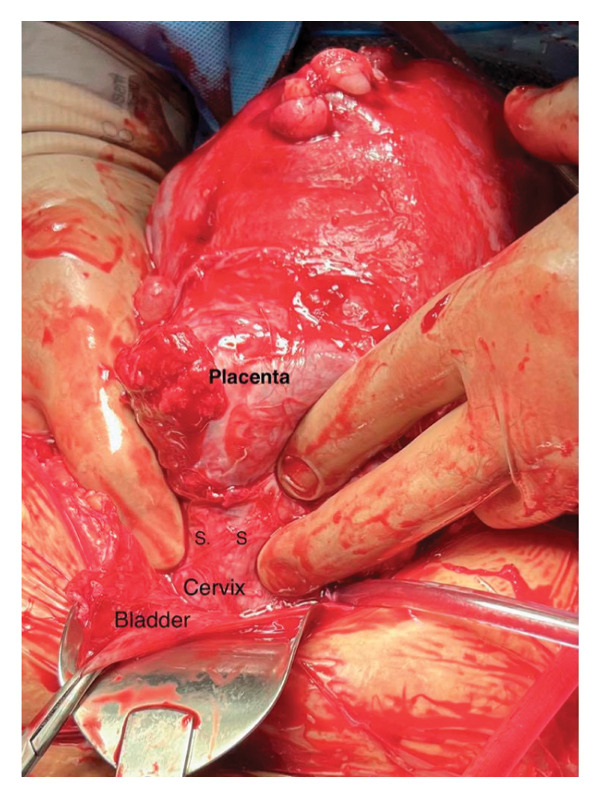
Intraoperative image showing the anterior lower uterine segment with placenta increta. The bladder is dissected and retracted, exposing the cervix and surgical site (S.S.) targeted for devascularization.

**FIGURE 2:**
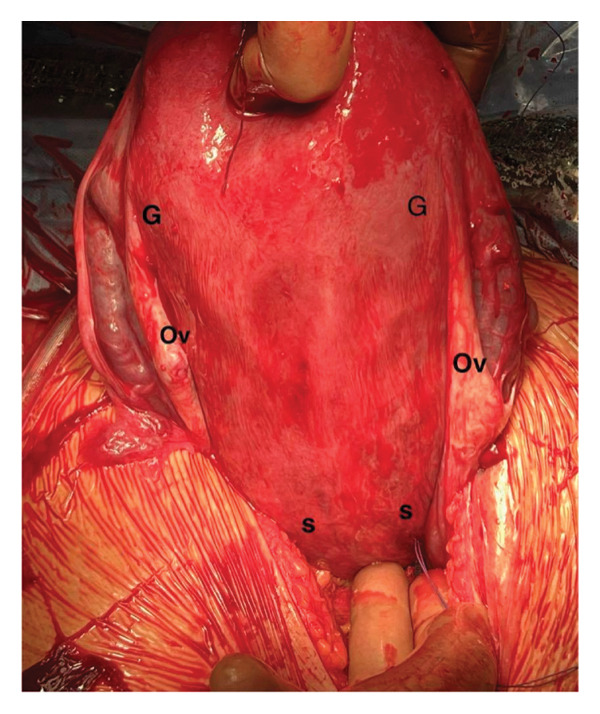
Posterior view of the uterus: (S) marks the exit of the SG suture at the uterosacral ligament, and (G) marks the sites of reinsertion of the SG suture below the insertion of the ovarian ligament.

**FIGURE 3:**
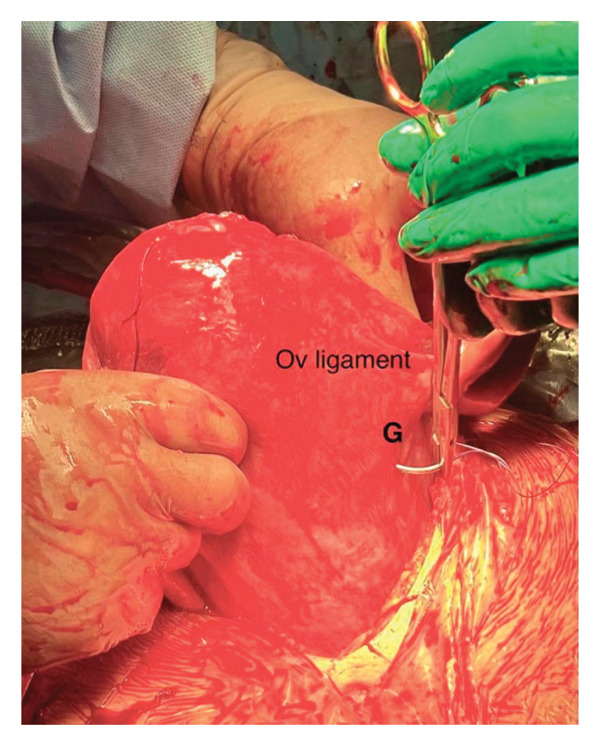
SG suture is applied under the ovarian ligament to ligate the lateral uterine blood supply.

**FIGURE 4:**
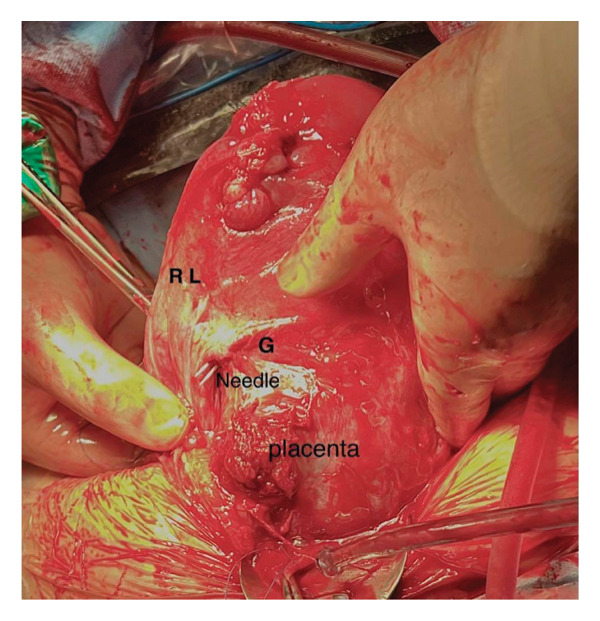
Anterior uterine wall showing the exit of SG suture below the insertion of the round ligament (RL).

**FIGURE 5:**
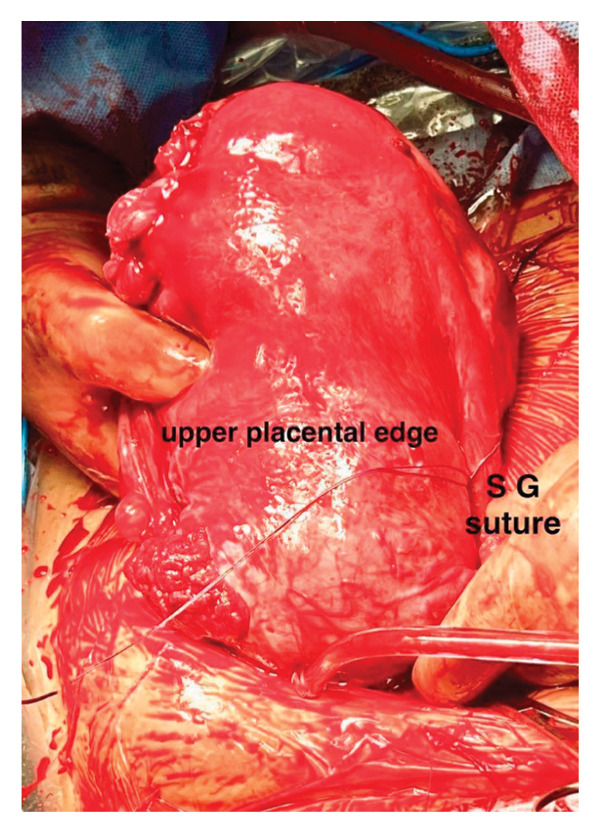
Uterus is tilted to the right to illustrate the left SG suture placement.

**FIGURE 6:**
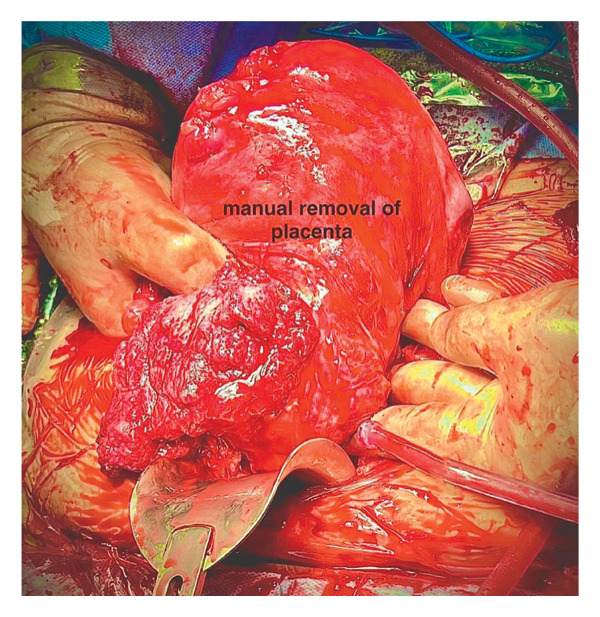
After applying both SG sutures on the lateral wall of the uterus, the placenta was removed manually.

**FIGURE 7:**
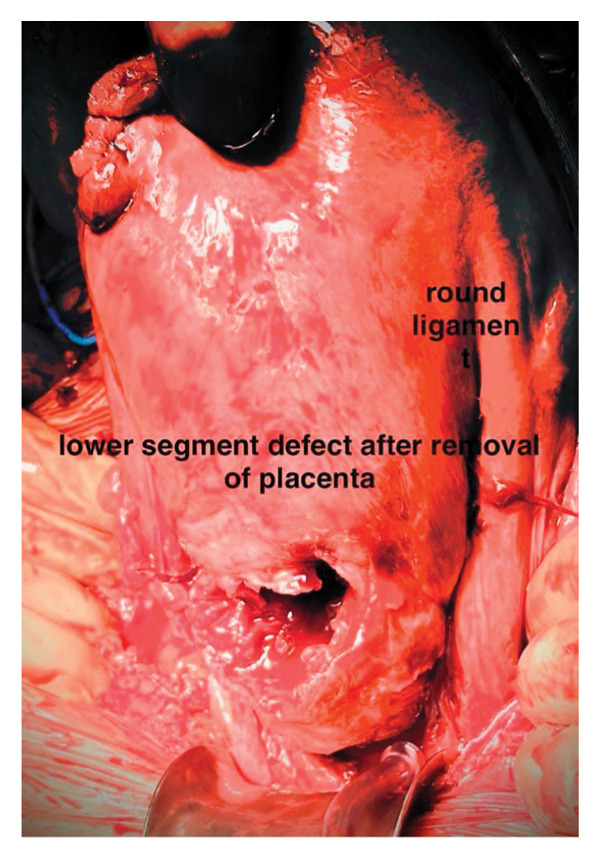
Anterior wall defect in the uterus after removal of the placenta.

**FIGURE 8:**
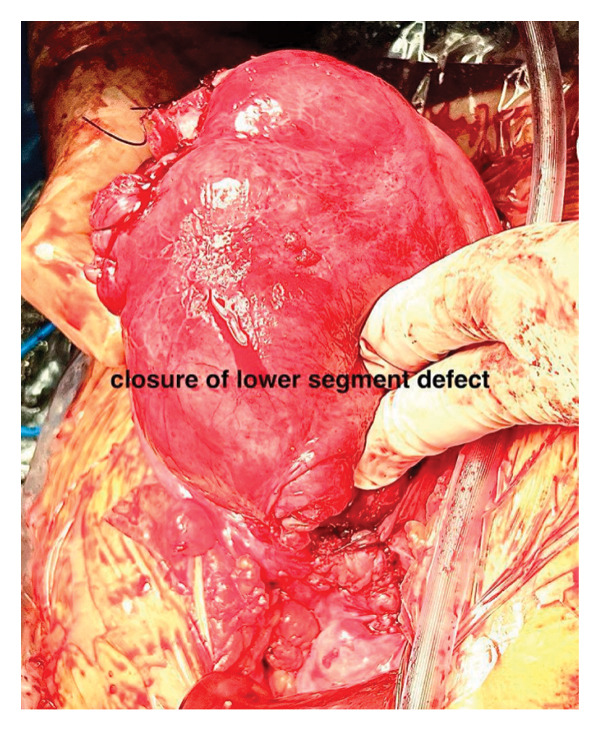
The uterus appearance after closure of the uterine wall defect.

**FIGURE 9:**
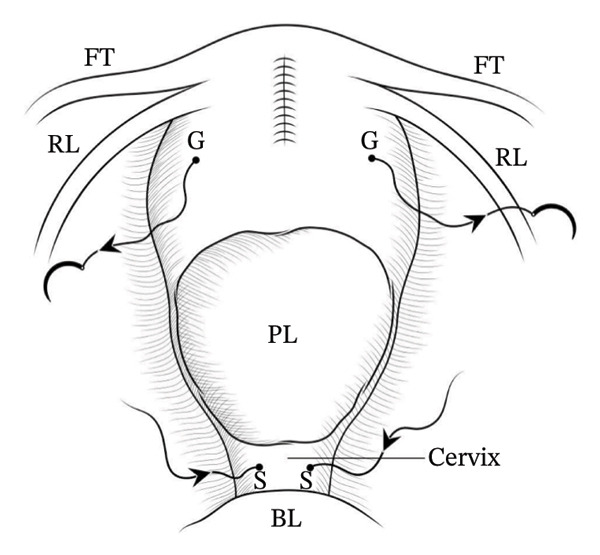
Anterior wall of the uterus, FT: fallopian tube, RL: round ligament, PL: placenta, BL: bladder, S is cervical entrance point, and G is the exit point of SG stitch.

**FIGURE 10:**
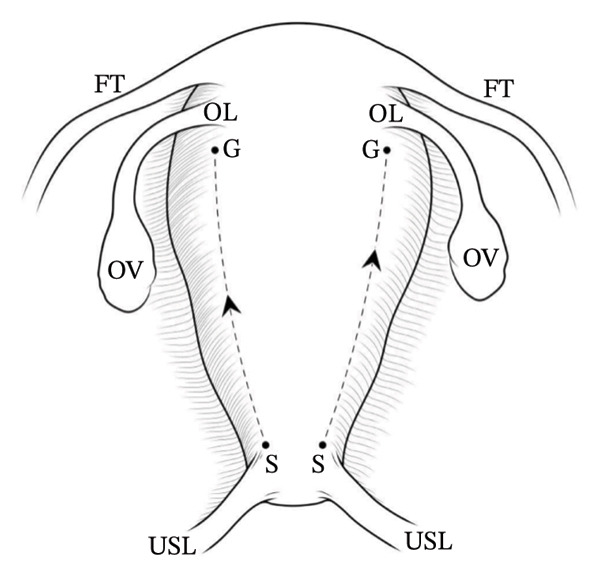
Posterior wall of the uterus, USL: uterosacral ligament, S is the exit of the suture, and G is the entrance of SG suture. OL: ovarian ligament, OV: ovary.

1. The abdomen is opened via a midline subumbilical incision. Uterine adhesions (except those at the placental site) are dissected to mobilize the uterus.
2. A midline upper segment uterine incision is made 3–5 cm above the upper placental edge; bleeding encountered from the uterine incision is the least if it is done precisely at midline.
3. The fetus is delivered; administration of uterotonics is avoided, as it may cause placental separation in the part of the placenta attached to the upper uterine segment.
4. The umbilical cord is cut at the placental insertion to allow maximum drainage of fetal placental blood, as decreasing the volume of the placental bulge may improve the operative field and facilitate adhesion dissection.
5. Once uterus is exteriorized, the adhesions over the placental site are dissected carefully and slowly using blunt and sharp dissection until the cervix is clearly visible, this step is the most critical part of surgery(If adhesions is obscuring the bladder landmarks, the bladder is filled through double lumen catheter with 300 cc of methylene blue in saline to define its border). Yet, in case of a severe form of PAS, bladder injury is difficult to avoid.
6. Then the membranes with the placenta are peeled off from the incision made previously. The incision sides are first to be sutured in two layers with Vicryl 1 in a big needle (VCP371).
7. The cervix is then stabilized manually by the assistant surgeon, two points at the cervix for entry of the vertical devascularization sutures are identified, the “S points” (the S points are located at least 1 cm cm below lower placental edge and 1 cm medial to the each lateral cervical borders), (Figure 1) a Vicryl 2 suture in a big needle (VCP9246) is passed through the full cervical thickness of the cervix, emerging at the uterosacral ligament (Figure 2). It is then reinserted at point “G” (just below the insertion of the ovarian ligament to the uterus and about 2 cm away from lateral uterine borders (Figure 3) and its exit is at the anterior uterine wall below the insertion of round ligament (Figure 4), it should be at above the upper placental insertion,. The stitch is firmly tied using surgical knots up to 4 knots and making sure that all parts of the placenta are medial to the suture path; this is achieved by uplifting the uterus to the opposite side. The process is repeated bilaterally, with a second set of sutures for effective devascularization. (Figure 5)
8. After the two sets of devascularization sutures are inserted, the placenta with its covering of the very thin myometrium layer is manually removed, and the uterine defect is repaired with continuous Vicryl 1 suture in two layers (Figures 6–8), diagrammatic illustrations are shown in Figures 9 and 10.
9. Hemostasis is completely secured on the uterus and bladder bed. The bladder is filled with 300 cc of methylene blue in saline through the double‐lumen Folly’s catheter, and any bladder injury is repaired in two layers using continuous three‐zero Vicryl suture.

.

### 2.2. Statistical Analysis

Data were analyzed using SPSS Version 22. Continuous variables were reported as means ± SD or medians; categorical variables as counts and percentages. Comparisons were made using ANOVA, and a *p* value < 0.05 was considered statistically significant.

### 2.3. Patient Characteristics

Among the 67 patients, the mean age was 35.97 ± 4.39 years. Median age was 37 years; the population was multiparous (mean gravida 6.16; mean parity 4.03). Most pregnancies were singleton (mean 1.03 fetuses, SD = 0.17), mean GA at admission was 32.07 ± 2.74 weeks, and average number of prior caesarean sections was 3.01(SD = 1.58). Antepartum bleeding occurred in 34.3% of patients (n = 23), with a mean of 1.91 episodes (SD = 1.21) (Table 1).

**TABLE 1:** Characteristics of the patients (*N* = 67).

		Mean	SD	Median	Minimum	Maximum
Age		35.97	4.39	37.00	25.00	44.00

Gravida		6.16	2.48	6.00	2.00	11.00

Para		4.03	1.91	4.00	0.00	8.00

No. of fetuses		1.03	0.17	1.00	1.00	2.00

GA on admission		32.07	2.74	32.00	24.00	38.00

Hx of cesarean deliveries		3.01	1.58	3.00	0.00	7.00

Bleeding episodes (*N*, %)	Yes	23 (34.33)				
	No	44 (65.67)				

No of bleeding episodes		1.91	1.20	1.00	1.00	5.00

### 2.4. Operative and Maternal Outcomes

Mean GA at delivery was 33.72 weeks. Elective caesarean was performed in 70.1% and emergency in 29.9%. Estimated blood loss was 1.57 ± 0.81 L. Preoperative and postoperative hemoglobin values were 12.84 and 11.47 g/dL, respectively.

Transfusions were required in 82.1% (*n* = 55), PRBC were administered to 80.60% with a mean of 2.39 ± 1.43 units. Cell‐saver use was recorded in 16.4%, FFP in 32.8%, and platelets in 9.0%. Conservative management succeeded in 95.5% (64/67), with 3 patients requiring hysterectomy. Bladder injury occurred in 22.4%; no maternal deaths were reported, although long‐term follow‐up was not conducted for our patients, and routine ultrasound was not obtained, as our patients were asymptomatic. Two of our patients were referred with subsequent pregnancies, in which one of them had recurrence of PAS, and she had repeated conservative surgical management along with tubal ligation (Table 2).

**TABLE 2:** Maternal outcome measures (*N* = 67).

		Mean	SD	Median	Minimum	Maximum
GA at delivery		33.72	4.55	34.00	0.00	38.00

Hospital stay post‐op (in days)		4.22	2.62	3.00	2.00	12.00

Pre‐op hemoglobin		12.84	12.10	11.50	9.00	110.00

Post‐op hemoglobin		11.47	9.73	10.30	7.60	88.00

Estimated blood loss (in liters)		1.57	0.81	1.50	0.50	4.00

CS (*N*, %)	Elective	47 (40.15)				
	Emergency	20 (29.85)				

Need for transfusion (*N*, %)	Yes	55 (82.09)				
	No	12 (17.91)				

Type of blood products						

PRBC (*N*, %)		54 (80.60)				

Number of units		2.39	1.43	2.00	1.00	8.00

Cell saver (*N*, %)		11 (16.42)				

mL		479.09	292.46	500.00	133.00	987.00

FFP (*N*, %)		22 (32.84)				

Number of units		2.73	1.49	2.00	1.00	7.00

Platelet (*N*, %)		6 (8.96)				

Number of units		2.33	1.75	1.50	1.00	5.00

Birth weight		2297.79	472.92	2205.00	1190.00	3840.00

Conservative measures applied (*N*, %)	Yes	64 (95.52)				
	No	3 (4.48)				

Cesarean hysterectomy (*N*, %)	Yes	3 (4.48)				
	No	63 (94.03)				
	Unknown	1 (1.49)				

NICU admission (*N*, %)	Yes	41 (61.19)				
	No	9 (13.43)				
	Unknown	17 (25.37)				

Bladder injury (*N*, %)	Yes	15 (22.39)				
	No	52 (77.61)				

DIC (*N*, %)	Yes	1 (1.49)				
	No	66 (98.51)				

### 2.5. Neonatal Outcomes

Mean neonatal birth weight was 2297.8 ± 472.9 g. NICU admission was needed in 61.2% of cases, consistent with preterm delivery prevalence.

### 2.6. Temporal Trends

**FIGURE 11:**
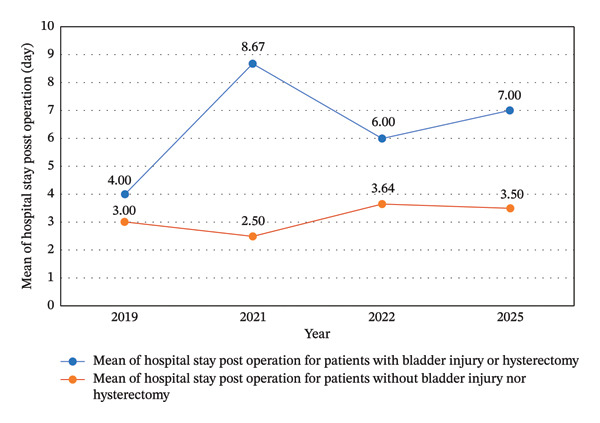
Mean of hospital stay postoperation for patients with bladder injury or hysterectomy and patients without bladder injury or hysterectomy.

**FIGURE 12:**
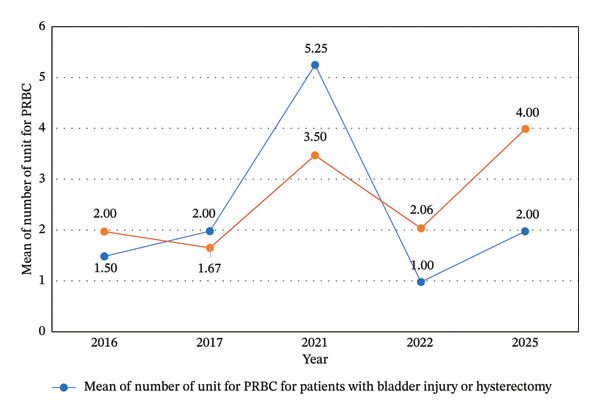
Mean number of units for PRBC.

**FIGURE 13:**
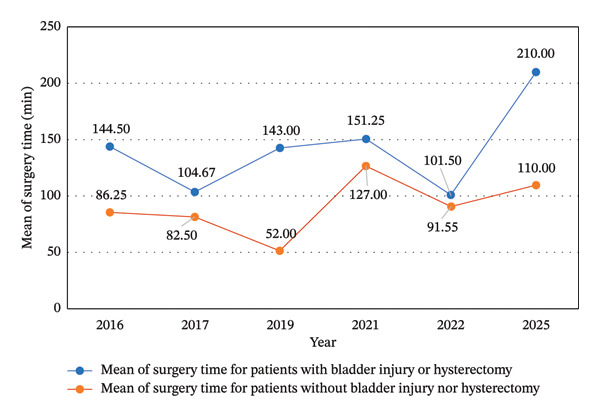
Mean of surgery time (minute).

Annual analysis demonstrated a statistically significant reduction in mean PRBC units transfused over time (*p* = 0.004), indicating improved hemostasis. Mean surgical time also differed significantly by year (*p* = 0.002), reflecting increased procedure efficacy with experience. Postoperative hospital stay did not differ significantly based on the presence of bladder injury or hysterectomy (*p* = 0.430 and 0.881, respectively). Indicating stable recovery outcomes across patient subgroups (Table 3 and Figures 11–13).

**TABLE 3:** Mean of number of units for PRBC, mean of hospital stay postoperation, and mean of surgery time.

Years	Mean of number of units for PRBC for patients		Mean of hospital stay postoperation for patients (day)		Mean of surgery time for patients (min)	
	With bladder injury or hysterectomy	Without bladder injury nor hysterectomy	With bladder injury or hysterectomy	Without bladder injury nor hysterectomy	With bladder injury or hysterectomy	Without bladder injury nor hysterectomy
2016	1.50	2.00			144.50	86.25
2017	2.00	1.67		2.00	104.67	82.50
2018	3.00		8.33		153.67	
2019	4.00		4.00	3.00	143.00	52.00
2020		2.00		3.00		69.00
2021	5.25	3.50	8.67	2.50	151.25	127.00
2022	1.00	2.06	6.00	3.64	101.50	91.55
2023		2.00		2.33		117.33
2024		2.00		3.33		96.33
2025	2.00	4.00	7.00	3.50	210.00	110.00
Total	3.07	2.13	7.56	3.11	139.06	97.67
*p* value	0.130		< 0.001^∗^		0.002^∗^	

^∗^The presence of bladder injury or hysterectomy is significant to the hospital stay and to the duration of surgery.

## 3. Discussion

Clinical criteria and sonographic feature of PAS was used for patient selection in this study, it has been reported that the most severe form of PAS cases occurs in women with anterior low lying placenta, < 2 cm from anterior os or placenta previa, and history of one or more cesarean in addition to the sonographic feature of PAS which include bridging vessels, placental bulge, thin myometrium, less than 1 mm, and loss of retro placental clear zone [1, 4].

Planned cesarean hysterectomy remains the most common method used to treat PAS [5, 6], but as it has been reported by different studies, the incidence of blood loss of more than 3500 mL is still high and alarming (62%) [7]. Disseminated intravascular coagulation may occur as a sequence to massive hemorrhage [8]. In a relatively recent randomized controlled trial that included 100 women with placenta previa and different grade of PAS, the use of intraoperative bilateral internal iliac artery balloon occlusion did not reduce the number of units of packed red blood cell transfused or otherwise improve outcomes [9] different strategies for management of massive intraoperative bleeding in a women with PAS, including internal iliac ligation, uterine devascularization and uterine compression sutures were proposed but there is no randomized control trial to prove their effectiveness [10]. Most adjunctive surgical approaches minimize intraoperative bleeding.

In PAS, aim to occlude one or more of the major uterine blood supply sources. However, the vascularity of the pregnant uterus affected by PAS is markedly increased due to neovascularization. The blood supply becomes extensive and may involve not only internal iliac arteries and ovarian blood vessels, but also contributions from the collateral may become significant during placental development and invasion, collateral may include, external ileovaginal, femoral as well as a collateral supply from surrounding pelvic tissues [11–13]. The novelty of our approach lies in its simplicity and the targeting devascularization of the specific uterine region most prone to severe hemorrhage during placental removal, effectively controlling bleeding regardless of the source of vascular blood supply.

The presence of a senior surgeon with expertise in the management of PA showed the strongest correlation with reduced morbidity [14–16]; the procedure of choice will have to be chosen according to operator experience and resources available. A reasonable approach suggests that the simplest technique with the lowest rate of complication should be performed first [17]. Most of the surgical techniques proposed previously were aiming in general to devascularize either the uterus or the pelvic supply to the uterus with or without resection and reconstruction of the involved uterine segment such as the triple P or the modified triple P [18–20]. However, Our technique targets the devascularization of all the placental implantation sites irrespective of the source of blood supply after reducing the sizes of placenta by draining most of fetal blood, which might be extremely helpful to achieve homeostasis in various circumstances especially in a situation where not all subspecialties available.

Targeted devascularization by SG suture is currently used in our obstetric unit successfully for indications other than PAS, such as in cases of uncontrolled placenta site bleeding, during removal of fibroid, and postpartum hemorrhage encountered at the time of cesarean section. Such further indications are currently investigated.

Our findings are particularly notable given the high rate of previous caesarean deliveries in the cohort (mean 3.01), which is a recognized risk factor for PAS. Despite this, most patients underwent successful conservative surgery, with acceptable blood loss and low rates of major complications.

This technique’s main strength is in the devascularization of the targeted part of the uterus which is likely to cause severe bleeding, allowing safe and effective placental removal without hysterectomy. Limitations include the single‐center design, absence of long‐term fertility follow‐up, and a lack of a control group. Larger multicenter studies are needed to validate outcomes and evaluate future reproductive performance.

## 4. Conclusion

Targeted devascularization of the lateral uterine wall by SG suture is a simple and promising uterus‐sparing approach, achieving high rates of uterine preservation; it is feasible and potentially beneficial in experienced hands. With experience, surgical performance, and outcomes improve significantly. The technique may serve as a conservative alternative to hysterectomy in most of the patients with PAS.

## Author Contributions

Samir Ghourab conceptualized the study, performed the surgeries, and contributed to manuscript drafting.

Osama Alomar assisted in surgical procedures, participated in patient follow‐up, and contributed to manuscript drafting.

Reem Labani conducted data collection, analysis and prepared the tables and figures, and contributed to manuscript drafting.

## Funding

No funding was received for this manuscript.

## Disclosure

All authors reviewed and approved the final version of the manuscript.

## Ethics Statement

Ethical approval for this study was obtained from the Institutional Review Board of King Faisal Specialist Hospital and Research Centre, Riyadh, Saudi Arabia (RAC number: 2131035). Informed consent was obtained from all participants prior to inclusion in the study. Written permission was also secured from the women whose placental images are included in this article.

## Conflicts of Interest

The authors declare no conflicts of interest.

## Supporting Information

Additional supporting information can be found online in the Supporting Information section.

## Supporting information


**Supporting Information** A Video h264‐720 is attached which highlight the main steps of the procedure.

## Data Availability

The data that support the findings of this study are available on request from the corresponding author. The data are not publicly available due to privacy or ethical restrictions.

## References

[1] Jauniaux E. R. , Alfirevic Z. , Bhide A. G. et al., Placenta Praevia and Placenta Accreta: Diagnosis and Management: Green-Top Guideline No. 27a, BJOG. (December 2018) 126, no. 1, e1–e48, 10.1111/1471-0528.15306. 30260097

[2] Jauniaux E. , Hussein A. M. , Fox K. A. , and Collins S. L. , New Evidence-Based Diagnostic, and Management Strategies for Placenta Accreta Spectrum Disorders, Best Practice & Research Clinical Obstetrics & Gynaecology. (November 2019) 61, 75–88, 10.1016/j.bpobgyn.2019.04.006. 31126811 PMC6929563

[3] van Beekhuizen H. J. , Stefanovic V. , Schwickert A. et al., A Multicenter Observational Survey of Management Strategies in 442 Pregnancies With Suspected Placenta Accreta Spectrum, Acta Obstetricia et Gynecologica Scandinavica. (March 2021) 100, no. S1, 12–20, 10.1111/aogs.14096. 33483943 PMC8048500

[4] Self A. , Cavallaro A. , and Collins S. L. , Placenta Accreta Spectrum: Imaging and Diagnosis, The Obstetrician & Gynecologist.

[5] Jauniaux E. , Grønbeck L. , Bunce C. , Langhoff-Roos J. , and Collins S. L. , Epidemiology of Placenta Previa Accreta: A Systematic Review and Meta-Analysis, BMJ Open. (November 2019) 9, no. 11, 10.1136/bmjopen-2019-031193. PMC6858111 31722942

[6] Fitzpatrick K. E. , Sellers S. , Spark P. , Kurinczuk J. J. , Brocklehurst P. , and Knight M. , The Management and Outcomes of Placenta Accreta, Increta, and Percreta in the UK: A Population-Based Descriptive Study, BJOG: An International Journal of Obstetrics and Gynaecology. (January 2014) 121, no. 1, 62–71, 10.1111/1471-0528.12405. PMC3906842 23924326

[7] Schwickert A. , van Beekhuizen H. J. , Bertholdt C. et al., Association of Peripartum Management and High Maternal Blood Loss at Cesarean Delivery for Placenta Accreta Spectrum (PAS): A Multinational Database Study, Acta Obstetricia et Gynecologica Scandinavica. (March 2021) 100, no. S1, 29–40, 10.1111/aogs.14103. 33524163

[8] Ghourab S. , Abdomino-Pelvic Packing to Control Severe Hemorrhage Following Caesarean Hysterectomy, Journal of Obstetrics and Gynecology. (January 1999) 19, no. 2, 155–158, 10.1080/01443619965480. 15512258

[9] Chen M. , Liu X. , You Y. et al., Internal Iliac Artery Balloon Occlusion for Placenta Previa and Suspected Placenta Accreta: A Randomized Controlled Trial, Obstetrics & Gynecology. (May 2020) 135, no. 5, 1112–1119, 10.1097/aog.0000000000003792. 32282608

[10] Morlando M. and Collins S. , Placenta Accreta Spectrum Disorders: Challenges, Risks, and Management Strategies, International Journal of Women’s Health. (November 2020) 12, 1033–1045, 10.2147/ijwh.s224191. PMC7667500 33204176

[11] Adelusi B. , Moghraby S. , Ghourab S. et al., Massive Recurrent Vaginal Bleeding Following Abdominal Hysterectomy, Annals of Saudi Medicine. (September 1995) 15, no. 5, 522–524, 10.5144/0256-4947.1995.522. 17590655

[12] Nabhan A. E. , AbdelQadir Y. H. , Abdelghafar Y. A. et al., Therapeutic Effect of Internal Iliac Artery Ligation and Uterine Artery Ligation Techniques for Bleeding Control in Placenta accreta Spectrum Patients: A Meta-Analysis of 795 Patients, PLoS One. (2022) 17, no. 9. 10.3389/fsurg.2022.983297 PMC9474733 36117806

[13] Barinov S. V. and Di Renzo G. C. , A New Technique to Preserve the Uterus in Patients With Placenta Accreta Spectrum Disorders, American Journal of Obstetrics and Gynecology. (2024) 230, no. 3 Suppl, S1107–S1115, 10.1016/j.ajog.2023.07.012. 37661498

[14] Erfani H. , Fox K. A. , Clark S. L. et al., Maternal Outcomes in Unexpected Placenta Accreta Spectrum Disorders: Single-Center Experience With a Multidisciplinary Team, American Journal of Obstetrics and Gynecology. (October 2019) 221, no. 4, 337-e1–337.e5, 10.1016/j.ajog.2019.05.035. PMC8651298 31173748

[15] Eller A. G. , Bennett M. A. , Sharshiner M. et al., Maternal Morbidity in Cases of Placenta Accreta Managed by a Multidisciplinary Care Team Compared With Standard Obstetric Care, Obstetrics & Gynecology. (February 2011) 117, no. 2 Part 1, 331–337, 10.1097/aog.0b013e3182051db2. 21309195

[16] Shamshirsaz A. A. , Fox K. A. , Erfani H. et al., Outcomes of Planned Compared with Urgent Deliveries Using a Multidisciplinary Team Approach for Morbidly Adherent Placenta, Obstetrics & Gynecology. (February 2018) 131, no. 2, 234–241, 10.1097/aog.0000000000002442. 29324609

[17] Collins S. L. , Alemdar B. , van Beekhuizen H. J. et al., Evidence-Based Guidelines for the Management of Abnormally Invasive Placenta: Recommendations From the International Society for Abnormally Invasive Placenta, American Journal of Obstetrics and Gynecology. (June 2019) 220, no. 6, 511–526, 10.1016/j.ajog.2019.02.054. 30849356

[18] Tskhay V. B. , Yametov P. K. , and Yametova N. M. , The Use of Modified Triple-P Method With Adherent Placenta Long-Term Results, MO Womens Health. (2017) 4, 30–32, 10.15406/mojwh.2017.04.00079.

[19] Ghaleb M. M. , Safwat S. , Purohit R. , and Samy M. , Conservative Stepwise Surgical Approach for Management of Placenta Previa Accreta: A Prospective Case Series Study, International Journal of Gynecology & Obstetrics. (2022) 157, no. 2, 383–390, 10.1002/1jgo.13887. 34549822

[20] Palacios-Jaraquemada I. M. , Fiorillo A. , Hamer I. , Martínez M. , and Bruno C. , Placenta Accreta Spectrum: A Hysterectomy can be Prevented in Almost 80% of Cases Using a Resective-Reconstructive Technique, Matern Fetal Neonatal Med.(2022) 35, no. 2, 275–282, 10.1080/14767058.2020.1716715. 31984808

